# The Influence of Anti-C3aR and Anti-C5aR Antibody Levels on the Course of Specific Glomerulonephritis Types

**DOI:** 10.3390/jcm14176082

**Published:** 2025-08-28

**Authors:** Maciej Szymczak, Harald Heidecke, Marcelina Żabińska, Łucja Janek, Jakub Wronowicz, Krzysztof Kujawa, Kai Schulze-Forster, Karolina Marek-Bukowiec, Tomasz Gołębiowski, Mirosław Banasik

**Affiliations:** 1 Clinical Department of Nephrology, Transplantation Medicine and Internal Diseases Wroclaw Medical University, 50-556 Wroclaw, Poland; karolina.marek-bukowiec@umw.edu.pl (K.M.-B.); tomasz.golebiowski@umw.edu.pl (T.G.); miroslaw.banasik@umw.edu.pl (M.B.); 2 CellTrend Gmbh, Im Biotechnologiepark 3 TGZ II, 14943 Luckenwalde, Germany; heidecke@celltrend.de (H.H.); schufo@celltrend.de (K.S.-F.); 3 Department of Preclinical Sciences, Pharmacology and Medical Diagnostics, Faculty of Medicine, Wrocław University of Science and Technology, 58-376 Wroclaw, Poland; marcelina.zabinska@pwr.edu.pl; 4 Statistical Analysis Center, Wroclaw Medical University, 50-368 Wroclaw, Poland; lucja.janek@umw.edu.pl (Ł.J.); jakub.wronowicz@umw.edu.pl (J.W.); krzysztof.kujawa@umw.edu.pl (K.K.)

**Keywords:** glomerulonephritis, complement 3a receptor, complement 5a receptor, antibodies

## Abstract

**Background**: The complement system factors’ role in the pathogenesis of autoimmunological diseases is known, but the influence of autoantibodies against complement factors’ receptors on the course of specific glomerular diseases remains unclear. **Methods**: We measured the levels of anti-C3aR and anti-C5aR antibodies in patients with membranous nephropathy (*n* = 18), primary focal and segmental glomerulosclerosis (FSGS) (*n* = 25), lupus nephritis (LN) (*n* = 17), IgA nephropathy (*n* = 14), mesangial proliferative (non-IgA) glomerulonephritis (*n* = 6), c-ANCA (cytoplasmic anti-neutrophil cytoplasmic antibodies) vasculitis (*n* = 40), and p (perinuclear)-ANCA vasculitis (*n* = 16). These conditions were compared to a healthy control group (*n* = 22). Then, for up to two years, we tracked the patients’ clinical progress (in terms of creatinine, total protein, and albumin levels) and compared the outcomes with their antibody levels. **Results**: The lupus nephritis group had higher levels of anti-C3aR and anti-C5aR antibodies than the other groups. The lupus nephritis group’s anti-C3aR antibody level showed a negative correlation with albumin and total protein at several time points of observation. Additionally, at numerous observational points, the anti-C3aR antibody level showed a positive correlation with both the basic albumin level in the FSGS group and the total protein level. **Conclusions**: The anti-C3aR and anti-C5aR antibodies are higher in lupus nephritis patients compared to other glomerulonephritis patients and healthy individuals. Albumin and total protein levels appear to be correlated positively with anti-C3aR antibody levels in FSGS and negatively in lupus nephritis.

## 1. Introduction

C3aR (receptor of complement factor 3a) is a kind of G-protein-coupled receptor (GPCR) that is expressed on cellular surfaces [1]. C3aR is involved in the development of inflammation [2], sepsis [3], arthritis [4], allergic asthma [5], metabolic dysfunction [6], diabetes [7], ischemia-reperfusion injury [8], neoplastic diseases [9], and morphogenesis [10]. C3aR signaling in kidney diseases was found to be involved in the pathogenesis of membranous nephropathy [11], focal and segmental glomerulosclerosis [12], lupus [13], IgA nephropathy [14], p-ANCA (cytoplasmic anti-neutrophil cytoplasmic antibodies) vasculitis [15], C3 glomerulopathy [16], and membrano-proliferative glomerulonephritis [17].

The mechanisms of C3aR involvement in specific kidney diseases include the following:

- Podocyte injury and glomerular membrane leakage in membranous nephropathy [11];
- Decay-accelerating factor expression diminishment in podocytes enhances C3 convertase, which activates C3aR [12]. C3a/C3aR ligation on podocytes [12] activates the interleukin-1beta/interleukin 1 receptor signaling loop, which results in a reduction in nephrin expression in focal and segmental glomerulosclerosis;
- Versican 1 expression activated by C3aR through the AKT/beta cathenin pathway in tubular cells induces fibroblast activation and, as a result, interstitial fibrosis [18] in focal and segmental glomerulosclerosis;
- The diminishment of C3aR expression ameliorates changes in experimental lupus [19]. C3aR expression was found particularly in the immunohistochemical staining results of kidney specimens from patients with active class IV lupus nephritis [13];
- C3aR caused cell proliferation and inflammation propagation through IL-6 (interleukin-6) and MCP-1 (monocyte chemoattractant protein 1) production enhancement in the course of IgA nephropathy [14];
- C3aR promotes macrophage infiltration in p-ANCA vasculitis but does not promote lymphocyte T activation [15];
- Widespread C3 glomerular deposition activates the complement system, including C3aR in C3 glomerulopathy [16];
- C3 convertase dysregulation leads to inflammation development in membranoproliferative glomerulonephritis [17].

Anti-C3aR antibodies are naturally occurring antibodies. They are present in healthy people like other anti-GCPR antibodies, and their levels can be changed over the course of the diseases. They modulate the activity of their receptors [20]. Their levels were found to be diminished in patients with Sjogren syndrome and rheumatoid arthritis compared to a healthy control group [21].

C5aR (receptor of complement factor 5a) is also a kind of GPCR [22]. C5aR is connected with the development of inflammation [23], sepsis [24], lupus [25], hypersensitivity arthritis [26], and neoplastic diseases [27].

C5aR is involved in the pathogenesis of kidney diseases including focal and segmental glomerulosclerosis [28,29], lupus nephritis [25,30], IgA nephropathy [31,32], c-ANCA vasculitis [33], p-ANCA (perinuclear anti-neutrophil cytoplasmic antibodies) vasculitis [34], ANCA-induced crescentic glomerulonephritis [35], C3 glomerulopathy [36], membranoproliferative glomerulonephritis [17], hemolytic uremic syndrome [37], and acute kidney injury [38].

The mechanisms of C5aR involvement in specific diseases include the following:

- Sphingosine kinase 1 pathway activation promotes inflammation development [28], the activation of parietal epithelial cells, and the mediation of podocyte injury [29] in focal and segmental glomerulosclerosis;
- Increasing Il-1 beta and MIP-2 mRNA (mitochondrial ribonucleic acid) expression encourages the development of inflammation and apoptosis enhancement [25], promoting mitochondrial fission and dysfunction in podocytes, which results in podocytes injury [30] and the modulation of lymphocytes’ Th1 responses in lupus nephritis [39];
- Mesangial cells’ proliferation is promoted and inflammation is activated through Il-6 and MCP-1 [31] in IgA nephropathy;
- C5aR plays a role in amplification loop and neutrophil priming [35] in the development of ANCA vasculitis;
- A deficiency of C5aR ameliorates pANCA vasculitis [34];
- A C5aR deficiency reduces C3 glomerulopathy severity [36];
- C5a convertase regulation disorders enhance inflammation development in membranoproliferative glomerulonephritis [17];
- C5aR is connected with vascular thrombosis in hemolytic uremic syndrome [37];
- C5aR plays a role in lipopolysaccharide-induced kidney injury through N-acetyl-β-D-glucosaminidase activation, ferroptosis, and mitochondrial damage [38].

Anti-C5aR antibodies are also naturally occurring antibodies. There are two types: anti-C5aR1 and anti-C5aR2 [40]. They are like anti-C3aR antibodies, which are anti-GPCR antibodies with similar characteristics [20]. Anti-C5aR antibodies were postulated to be antagonistic to their receptor [41]. In our study, we assessed classical anti-C5aR1 according to other GPCR antibody evaluation methods [42].

Anti-C5aR antibody levels were found to be decreased in c-ANCA vasculitis compared to healthy controls [41]. Levels of anti-C5aR antibody were also connected with relapses in patients with eosinophilic granulomatosis with polyangiitis [43]. Decreased anti-C5aR antibody levels were also noted in patients with Sjogren syndrome [21].

Our study’s objectives were to assess the levels of anti-C3aR and anti-C5aR antibodies in various glomerular disorders, look for correlations between these antibodies and clinical information, and determine whether these antibodies would be a good fit for future research assessing their predictive value over the course of particular glomerular diseases.

The current study assessed the connections between levels of anti-C3aR and anti-C5aR antibodies in glomerular disorders. Additionally, we monitored patient clinical outcomes for two years after the antibody assessment to look into the relationship between baseline antibody concentrations and the clinical development of particular diseases. We planned to check the usefulness of these antibodies as a marker of glomerular disease course.

## 2. Materials and Methods

**Figure 1:**
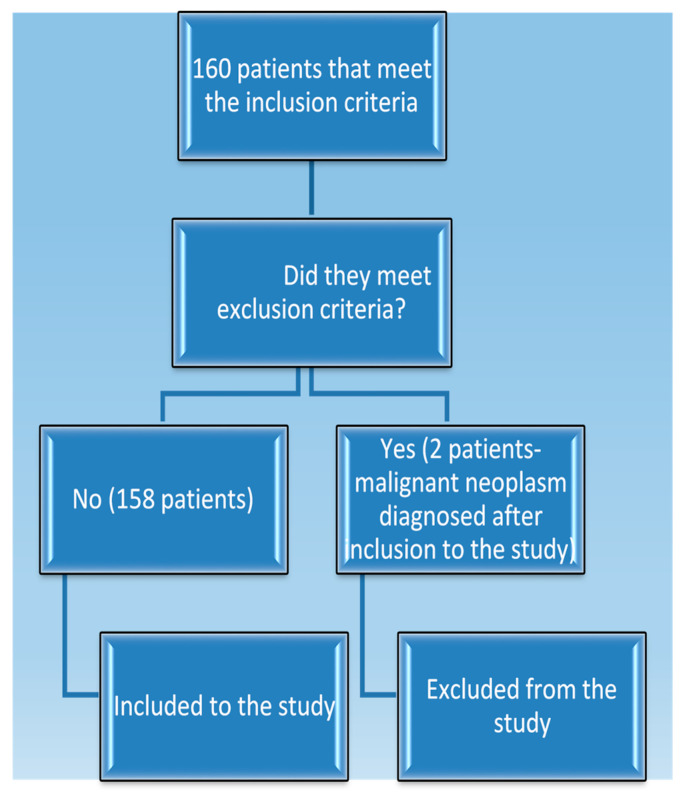
A flowchart outlining the procedures for inclusion of participants in the study.

Patients with the earliest signs of illness kidney disease or recurrence who visited our clinic between 2013 and 2020 were enrolled in the trial prior to starting immunosuppressive therapy (such as cyclophosphamide and steroid pulses) and before starting ACE (angiotensin converting enzyme) inhibitor treatment. At the time of enrollment or during the preceding six months, none of the study participants received any medication. Because of the design of the trial, there was no randomization. Only patients with a histopathologically confirmed diagnosis of either cytoplasmic (c) or perinuclear (p) positive cytoplasmic antibodies against neutrophils (ANCA) vasculitis, IgA (immunoglobulin A) nephropathy, non-IgA mesangial proliferative glomerulopathy, primary FSGS (focal and segmental glomerulosclerosis), or membranous nephropathy were included in the study. Additionally, the patients had to have proteinuria (at least 60 mg per day) at the time of their most recent (one or two days before material collection) available urine evaluation in order to be included. The study excluded patients with active infections, dialysis, kidney transplants, or a history of cancer, either past or present. All these diagnoses can modulate the immunological system and influence the antibody level results. A total of 22 healthy people of Caucasian descent who satisfied the following requirements made up the control group: they had no history of kidney, autoimmune, or neoplastic problems in anamnesis. Their CRP levels were less than 5 mg/dL, their creatinine levels were not higher than 1.3 mg/dL, and they did not have proteinuria or erythrocyturia. Control group consisted of workers at our clinic, and their inclusion was consecutive and random without additional selection criteria apart from those mentioned above. A flowchart showing the steps involved in including study participants is shown in Figure 1.

All the study participants were from Poland, and all of them signed an informed consent form prior to the materials being gathered. The Bioethics Committee of Wroclaw Medical University approved the study under numbers KB-221/2023 and KB-546/2012.

A total of 158 healthy controls and patients with the following glomerular diseases had their serum collected: membranous nephropathy (*n* = 18), focal and segmental glomerulosclerosis (FSGS) (*n* = 25), lupus nephritis (LN) (*n* = 17), IgA nephropathy (*n* = 14), non-IgA mesangial proliferative glomerulonephritis (*n* = 6), c-ANCA-positive vasculitis (*n* = 40), and p-ANCA-positive vasculitis (*n* = 16).

A second sample tube was used to collect an additional 2.7 mL of blood from each patient without the need for further venipuncture, in addition to the blood drawn for standard laboratory tests. There was no requirement for patients to refrain from eating on the morning prior to the blood draw. To avoid bias brought on by variations in storage duration prior to freezing, centrifugation of the samples started precisely ten minutes after collection. When the temperature rose above 28 °C, no blood samples were taken (according to the standards of our laboratory). The serum was isolated and kept at −80 °C after the blood was spun at 1500× *g* for 10 min.

Development of the techniques to detect anti-C3aR antibodies started many years ago [40], but the idea of detecting them as circulating autoantibodies appeared recently. The exact antibodies that we detected were anti-C3aR1. C3aR2 exists [44] but antibodies for this kind of receptor were not described.

The specific method of evaluation for these antibodies has been previously described, along with methods for evaluating other GPCR antibodies [42].

Using enzyme-linked immunosorbent assay (ELISA) kits received from Cell-Trend in Luckenwalde, Germany, the serum concentrations of anti-C3aR and anti-C5aR antibodies were determined according to the manufacturer’s guidelines: the anti-C3aR antibody kit and the anti-C5aR antibody kit. Microtiter plates were initially coated with C3aR and C5aR, followed by the addition of diluted samples (1:100), which were incubated at temperatures ranging from 2 to 8 °C for two hours. Horseradish peroxidase (POD)-conjugated anti-human IgG (1:100) was used to identify anti-C3aR and anti-C5aR antibodies following the washing procedures. This was followed by the development of a 3, 3′, 5, 5′-tetramethylbenzidine (TMB) substrate solution. The optical density was consequently translated into a concentration via a standard curve after measurements were taken at 450 nm with a correction according to 630 nm wavelength. A value of ≥2.5 U/mL was deemed positive, while a value of <2.5 U/mL was classified as negative for both anti-C3aR and anti-C5aR antibodies. The ELISA (enzyme-linked immunosorbent assay) procedures were validated in line with the FDA’s (Food and Drug Administration) Bioanalytical Method Validation Guidance for Industry [45]. There were no significant cross-reactions with different lupus antibodies found.

Clinical information was gathered for serum creatinine, estimated glomerular filtration rate (eGFR) (MDRD formula—Modification of Diet in Renal Disease), blood urea nitrogen (BUN), proteinuria, urine albumin/creatinine ratio, total serum protein, and serum albumin at the beginning of the observation period, as well as at one and three months, half a year, one year, and two years, while age and sex were also documented.

The concentrations of anti-C3aR and anti-C5aR antibodies, serum creatinine levels, eGFR, BUN, serum albumin, total serum protein, levels of proteinuria, the albumin/creatinine ratio, age, and sex were compared between the glomerulonephritis group and the control group using the Kruskal–Wallis test, Dunn’s test adjusted with Bonferroni correction, or analysis of variance (ANOVA). Matching for age and sex was performed.

To analyze correlations between quantitative variables, Pearson’s or Spearman’s correlation coefficients were employed, preceded by a Shapiro–Wilk test to assess the distribution of the data. The significance of the correlations was evaluated using a *t*-test, with a *p*-value of less than 0.05 indicating a significant finding. *p*-values were corrected for multiple comparisons. This correlation analysis examined the relationship between anti-C3aR and anti-C5aR antibody levels and clinical data.

The levels of ANA (antinuclear antibodies) in lupus nephritis patients, c-ANCA levels in granulomatosis with polyangiitis patients, and p-ANCA levels in p-ANCA-positive vasculitis patients were further compared to the concentrations of anti-C3aR and anti-C5aR antibodies.

Based on the initial levels of anti-C3aR and anti-C5aR antibodies in each group, Spearman’s correlation was used to evaluate the variability of clinical markers (serum creatinine, eGFR, BUN, serum albumin, and serum total protein levels) throughout a two-year observation period. The statistical range, standard deviation, coefficient of variation, and trends in antibody levels over the course of the study were all evaluated as part of the analysis. STATISTICA 13 was used for all data analyses.

## 3. Results

### 3.1. Patient Clinical Data

The clinical measures showed no statistically significant differences among the groups, except for the following cases.

Creatinine levels were higher in patients with p-ANCA-positive vasculitis than in the control group (*p* < 0.05). The p-ANCA vasculitis groups exhibited lower eGFR and higher BUN levels than the other groups (*p* < 0.05). Patients in the control group exhibited higher albumin and total protein concentrations than those in the other groups (*p* < 0.05). Patients in the disease-specific groups had proteinuria, whereas those in the control group did not. The hematocrit level was lower in the c-ANCA vasculitis group than in the control group (*p* < 0.05).

The clinical data for the patients is shown in Table 1 and Table 2.

### 3.2. Anti-C3aR Antibody Evaluation Results

The median anti-C3aR antibody levels were as follows:

5.4 (range: 2.5–11.2) U/mL in the membranous nephropathy group;
6 (range: 2.8–15.3) U/mL in the FSGS group;
15.8 (range: 8.7–61) U/mL in the lupus nephritis group;
7.15 (range: 2.4–16.7) U/mL in the IgA nephropathy group;
5.85 (range: 2.3–8.2) U/mL in the mesangial proliferative (non-IgA) glomerulonephritis group;
6.75 (range:4.5–44) U/mL in the control group;
6.5 (range: 3.9–55.1) U/mL in the c-ANCA vasculitis group;
6.95 (range: 4.2–49.3) U/mL in the p-ANCA vasculitis group.

Out of all the patients, 99% had positive results and the remaining 1% had results close to the positive cutoff, so the positive and negative cutoffs are not further elaborated on in this study.

**Figure 2:**
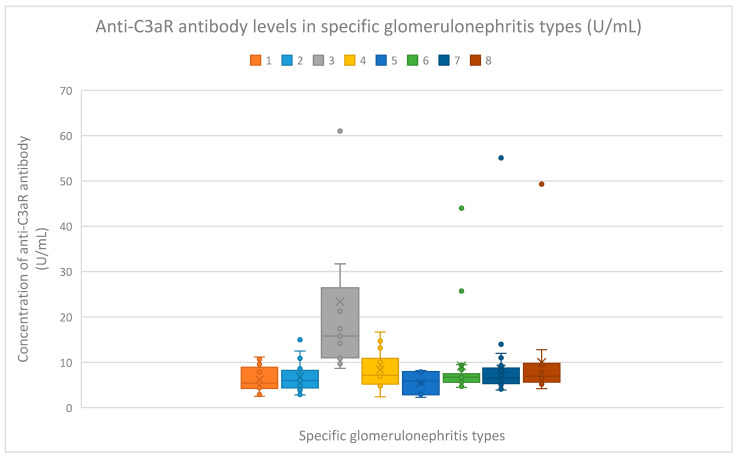
Anti-C3aR antibody levels (U/mL) in particular kinds of glomerulonephritis. The anti-C3aR antibody levels in patient groups 1–8 are represented by the bars with colors denoted by numbers 1–8. The antibody concentration (U/mL) is shown on the *y*-axis. The points represent patients. The median value is shown by X. 1—membranous nephropathy; 2—focal and segmental glomerulosclerosis; 3—lupus nephritis; 4—IgA nephropathy; 5—non-IgA mesangial proliferative glomerulonephritis; 6—the control group; 7—c-ANCA-positive vasculitis; 8—p-ANCA-positive vasculitis.

The lupus nephritis (*n* = 17) group had higher levels of serum anti-C3aR antibodies than all other groups, including the following: the membranous nephropathy group (*n* = 18) (*p* < 0.001), focal and segmental glomerulosclerosis group (*n* = 25) (*p* < 0.001), IgA nephropathy group (*n* = 14) (*p* = 0.01), mesangial proliferative (non-IgA) glomerulonephritis group (*n* = 6) (*p* = 0.002), control group (*n* = 22) (*p* < 0.001), c-ANCA vasculitis group (*n* = 40) (*p* < 0.001), and p-ANCA vasculitis group (*n* = 16) (*p* = 0.01). (Figure 2). Figure S1 presents the anti-C3aR antibody levels in distinct patients belonging to particular groups.

### 3.3. Anti-C5aR Antibody Evaluation Results

The median anti-C5aR antibody levels were as follows:

5.8 (range: 1.7–14.5) U/mL in the membranous nephropathy group;
7.1 (range: 2–13.7) U/mL in the FSGS group;
52.6 (range: 9.4–74.3) U/mL in the lupus nephritis group;
6.9 (range: 2–25) U/mL in the IgA nephropathy group;
6.4 (range: 1.3–8.2) U/mL in the mesangial proliferative (non-IgA) glomerulonephritis group;
5.35 (range: 3–47.4) U/mL in the control group;
5.55 (range: 2.7–17.1) U/mL in the c-ANCA vasculitis group;
8.05 (range: 2.9–56.1) U/mL in the p-ANCA vasculitis group.

Out of all the patients, 97% had positive results and the remaining 3% had results close to the positive cutoff, so the positive and negative cutoffs are not further elaborated on in this study.

**Figure 3:**
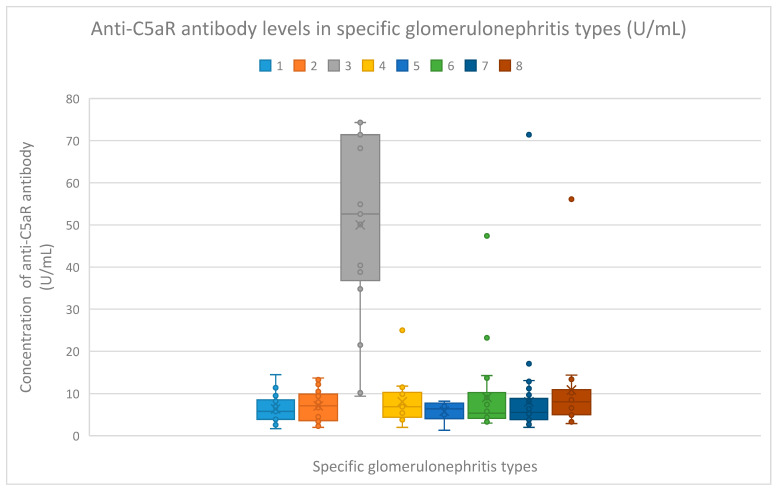
Anti-C5aR antibody levels (U/mL) in particular kinds of glomerulonephritis. The anti-C5aR antibody levels in patient groups 1–8 are represented by the bars with colors denoted by numbers 1–8. The antibody concentration (U/mL) is shown on the *y*-axis. Points represent patients. The median value is shown by X. 1—membranous nephropathy; 2—focal and segmental glomerulosclerosis; 3—lupus nephritis; 4—IgA nephropathy; 5—non-IgA mesangial proliferative glomerulonephritis; 6—the control group; 7—c-ANCA-positive vasculitis; 8—p-ANCA-positive vasculitis.

The serum anti-C5aR antibody levels in the membranous nephropathy group (*n* = 18) (*p* < 0.001), focal and segmental glomerulosclerosis group (*n* = 25) (*p* < 0.001), IgA nephropathy group (*n* = 14) (*p* = 0.002), mesangial proliferative (non-IgA) glomerulonephritis group (*n* = 6) (*p* = 0.008), control group (*n* = 22) (*p* < 0.001), c-ANCA vasculitis group (*n* = 40) (*p* < 0.001), and p-ANCA vasculitis group (*n* = 16) (*p* = 0.006) were all lower than the serum anti-C5aR antibody levels in the lupus nephritis group (*n* = 17) (Figure 3). The anti-C5aR antibody values in particular patients of specific groups are presented in Figure S2.

### 3.4. Relationships Between Patient Clinical Information and Anti-C3aR Antibody Levels

**Figure 4:**
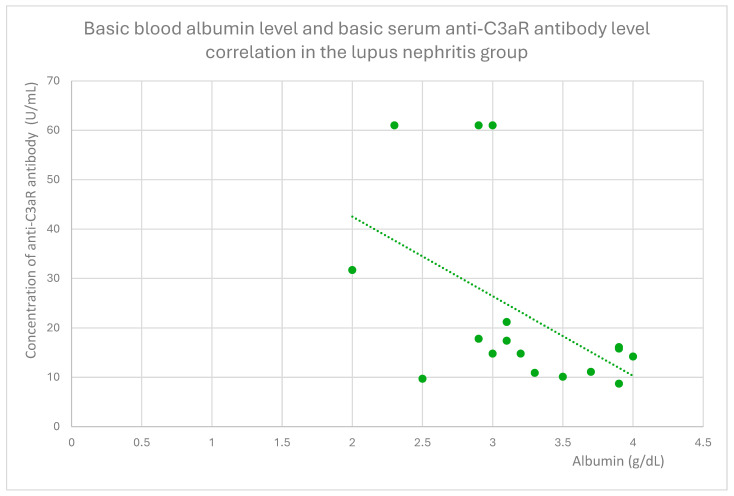
Basic serum albumin level and basic serum anti-C3aR antibody level were correlated in the lupus nephritis group (r = −0.54; *p* = 0.02). Specific patients are represented by green dots. The trend line is the green dotted line.

**Figure 5:**
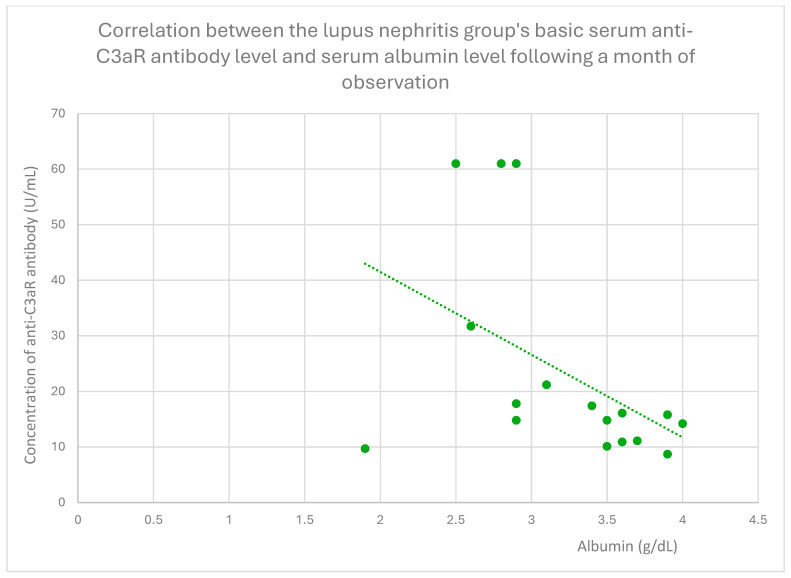
The lupus nephritis group showed a correlation between the basic serum anti-C3aR antibody level and serum albumin level after one month of monitoring (r = −0.51; *p* = 0.04). Specific patients are represented by green dots. The trend line is the green dotted line.

**Figure 6:**
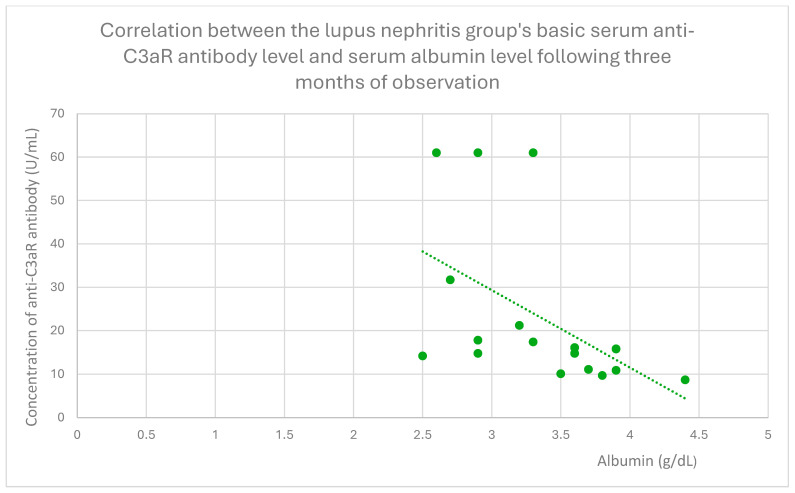
Correlation between the lupus nephritis group’s basic serum anti-C3aR antibody level and serum albumin level following three months of monitoring (r = −0.62; *p* = 0.01). Specific patients are represented by green dots. The trend line is the green dotted line.

**Figure 7:**
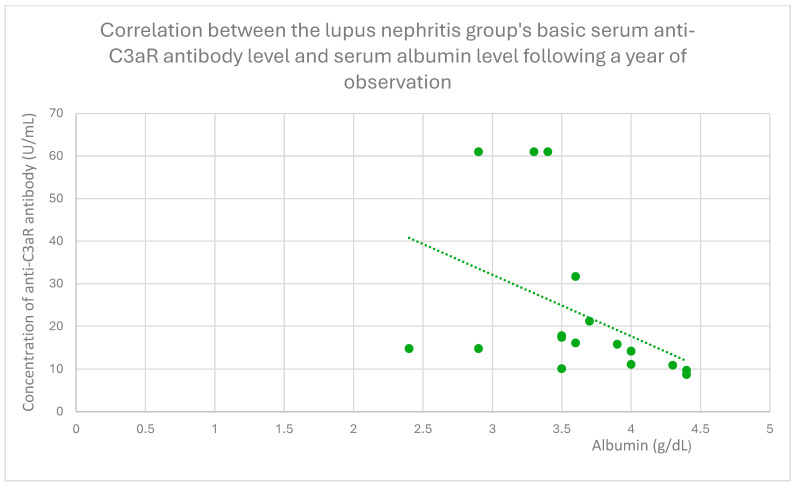
Correlation between basic serum anti-C3aR antibody level and serum albumin level after 12 months of observation in lupus nephritis group (r = −0.57; *p* = 0.03). Specific patients are represented by green dots. The trend line is the green dotted line.

**Figure 8:**
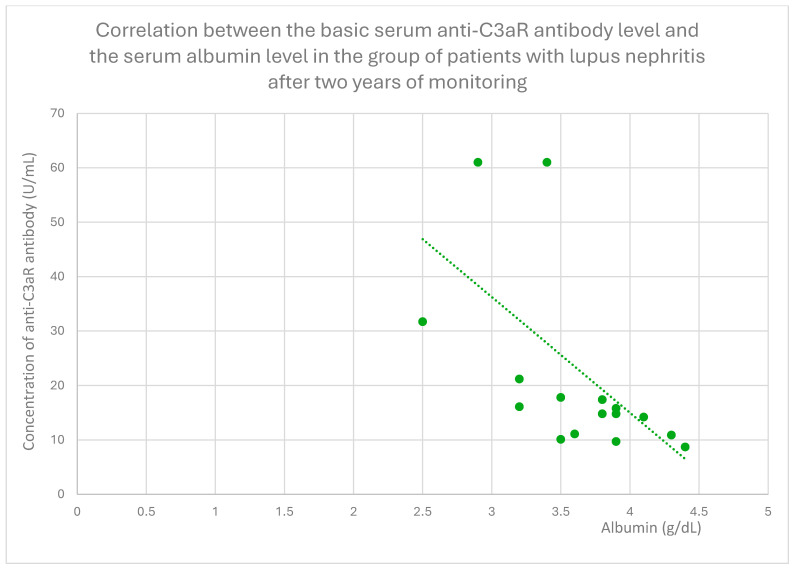
Correlation between the lupus nephritis group’s basic serum anti-C3aR antibody level and serum albumin level following 2 years of monitoring (r = −0.62; *p* = 0.01). Specific patients are represented by green dots. The trend line is the green dotted line.

**Figure 9:**
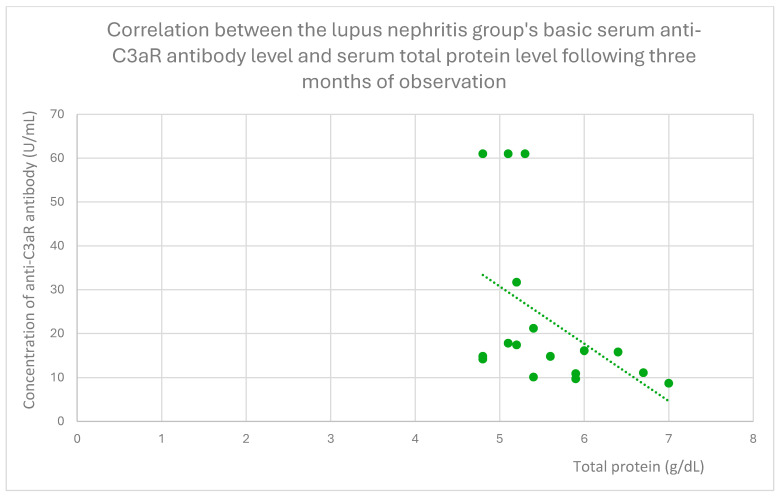
Correlation between the lupus nephritis group’s basic serum anti-C3aR antibody level and serum total protein level following three months of observation (r = −0.52; *p* = 0.04). Specific patients are represented by green dots. The trend line is the green dotted line.

**Figure 10:**
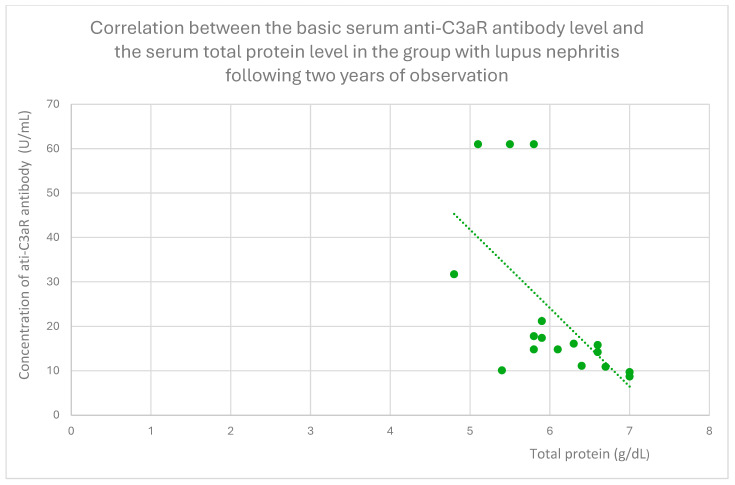
Correlation between the basic serum anti-C3aR antibody level and the serum total protein level in the group with lupus nephritis following two years of observation (r = −0.54; *p* = 0.02). Specific patients are represented by green dots. The trend line is the green dotted line.

**Figure 11:**
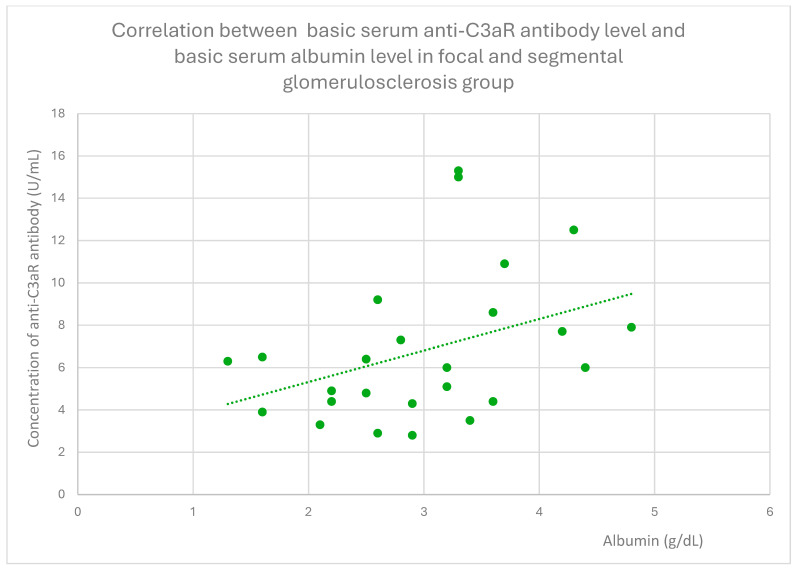
Correlation between basic serum anti-C3aR antibody level and basic serum albumin level in focal and segmental glomerulosclerosis group (r = 0.41; *p* = 0.03). Specific patients are represented by green dots. The trend line is the green dotted line.

The anti-C3aR baseline serum antibody level correlated negatively with serum albumin level at baseline (Figure 4) and after 1 month (Figure 5), 3 months (Figure 6), 12 months (Figure 7), and 2 years (Figure 8) of observation in the lupus nephritis group. Furthermore, after three months (Figure 9) and two years (Figure 10) of monitoring, the anti-C3aR antibody level in the same group showed a negative correlation with the serum total protein level. Moreover, the anti-C3aR antibody level in the lupus nephritis group correlated positively with the serum anti-dsDNA (anti-double-stranded deoxyribonucleic) antibody level (Figure 11).

**Figure 12:**
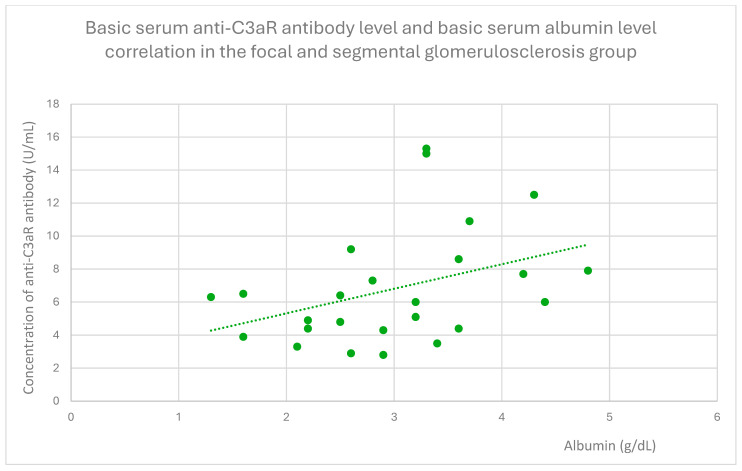
Basic serum anti-C3aR antibody level and basic serum albumin level correlation in the focal and segmental glomerulosclerosis group (r = 0.41; *p* = 0.03). Specific patients are represented by green dots. The trend line is the green dotted line.

**Figure 13:**
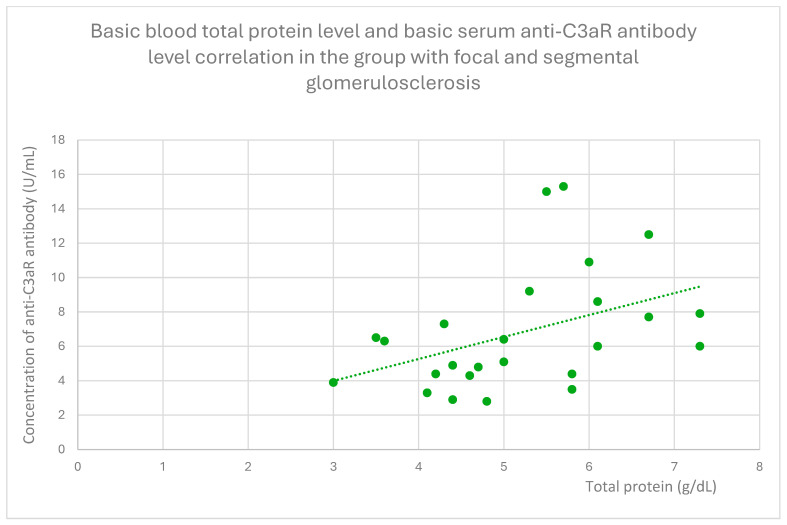
Basic blood total protein level and basic serum anti-C3aR antibody level correlation in the group with focal and segmental glomerulosclerosis (r = 0.46; *p* = 0.01). Specific patients are represented by green dots. The trend line is the green dotted line.

**Figure 14:**
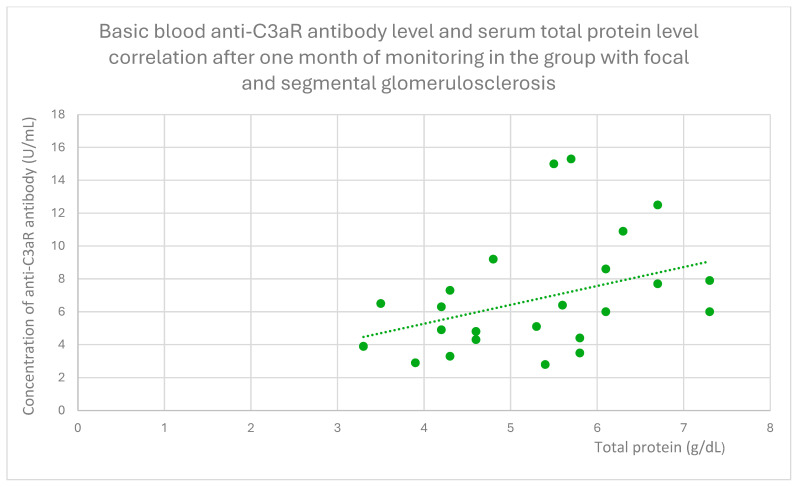
Basic blood anti-C3aR antibody level and serum total protein level correlation after one month of monitoring in the group with focal and segmental glomerulosclerosis (r = 0.51; *p* = 0.01). Specific patients are represented by green dots. The trend line is the green dotted line.

**Figure 15:**
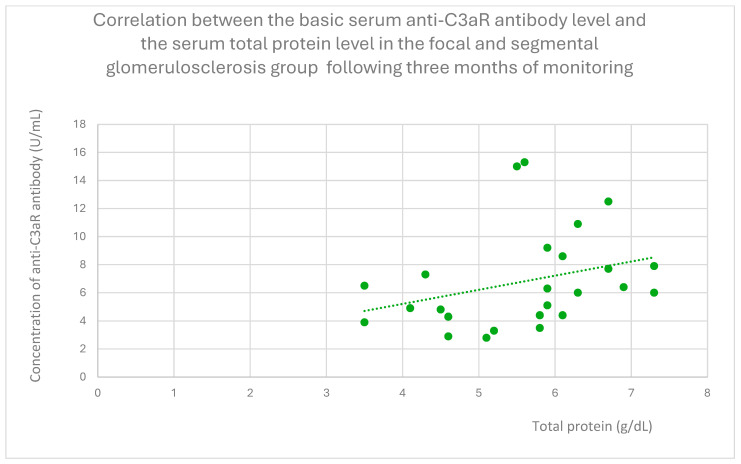
Correlation between the basic serum anti-C3aR antibody level and the serum total protein level in the focal and segmental glomerulosclerosis group following three months of monitoring (r = 0.48; *p* = 0.02). Specific patients are represented by green dots. The trend line is the green dotted line.

**Figure 16:**
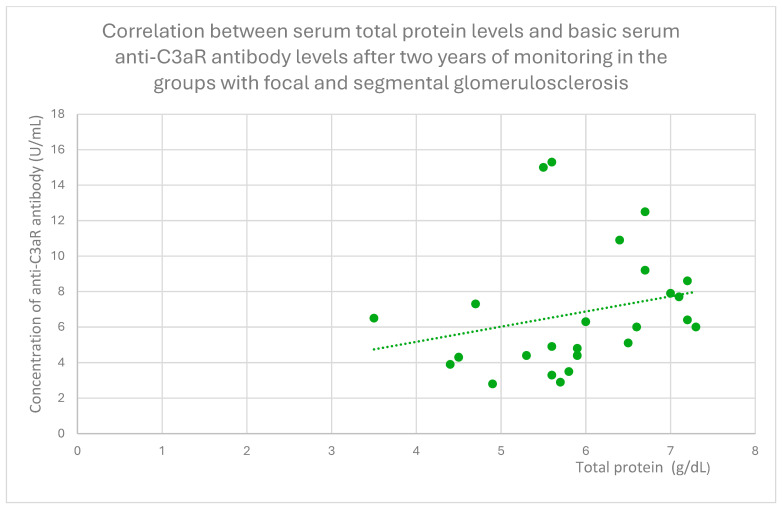
Correlation between serum total protein levels and basic serum anti-C3aR antibody levels after two years of monitoring in the groups with focal and segmental glomerulosclerosis (r = 0.48; *p* = 0.04). Specific patients are represented by green dots. The trend line is the green dotted line.

Anti-C3aR antibody levels also correlated positively with baseline albumin (Figure 12) and total protein levels at baseline (Figure 13) and after 1 month (Figure 14), 3 months (Figure 15), and 2 years (Figure 16) of observation in the focal and segmental glomerulosclerosis group.

**Figure 17:**
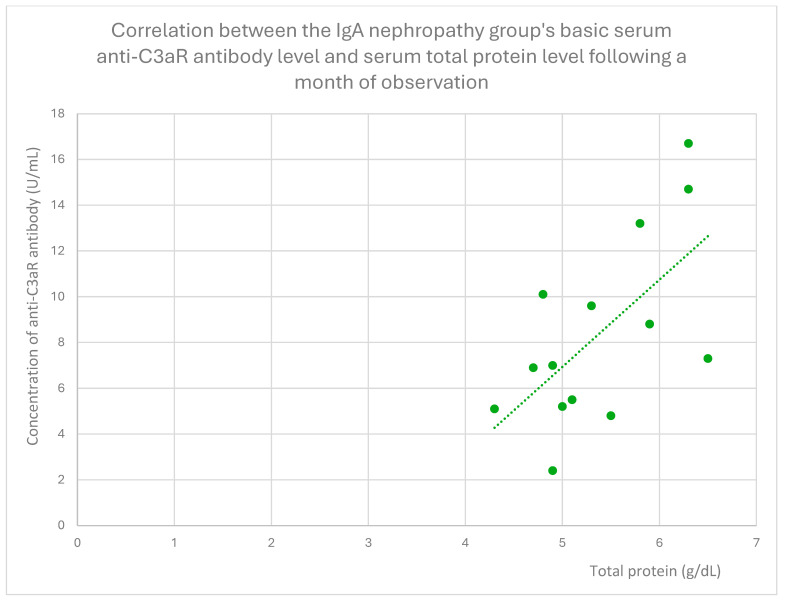
Correlation between the IgA nephropathy group’s basic serum anti-C3aR antibody level and serum total protein level following a month of observation (r = 0.53; *p* = 0.04). Specific patients are represented by green dots. The trend line is the green dotted line.

**Figure 18:**
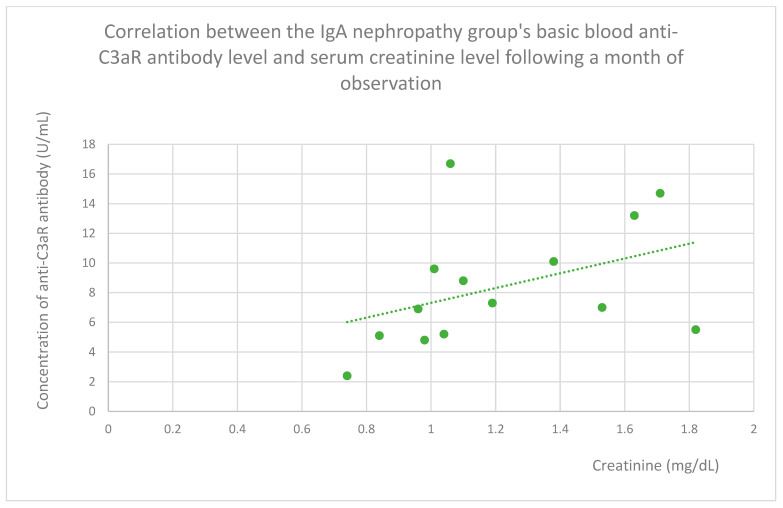
Correlation between the IgA nephropathy group’s basic blood anti-C3aR antibody level and serum creatinine level following a month of observation (r = 0.57; *p* = 0.03). Specific patients are represented by green dots. The trend line is the green dotted line.

Following a month of observation in the IgA nephropathy group, we also observed statistically significant positive relationships between anti-C3aR antibody levels and total protein (Figure 17) and creatinine (Figure 18).

### 3.5. Relationships Between Patient Clinical Data and Anti-C5aR Antibody Level

**Figure 19:**
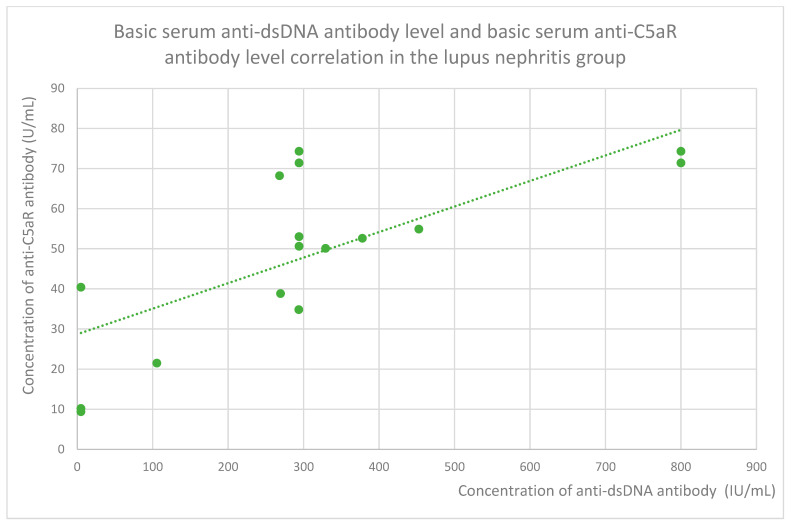
The correlation of basic serum anti-dsDNA antibody level and basic serum anti-C5aR antibody level in the lupus nephritis group (r = 0.76; *p* = 0.001). Specific patients are represented by green dots. The trend line is the green dotted line.

**Figure 20:**
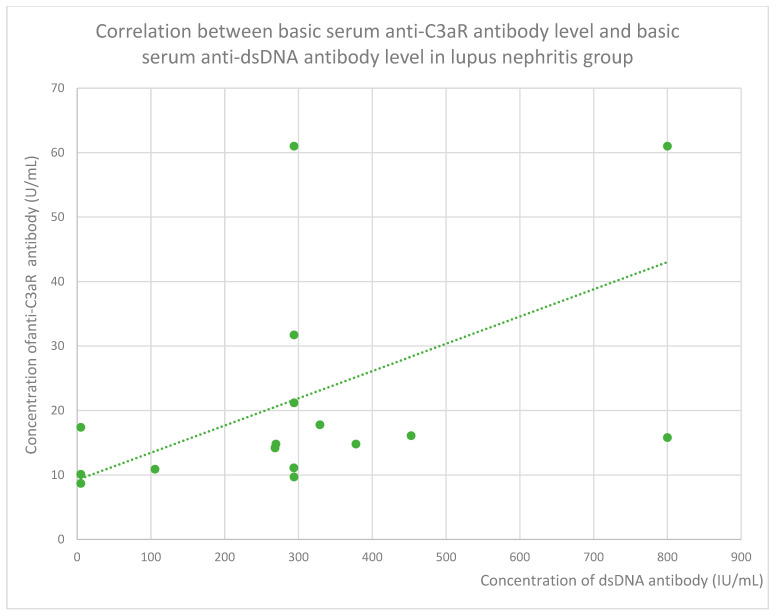
Correlation between basic serum anti-C3aR antibody level and basic serum anti-dsDNA antibody level in lupus nephritis group (r = 0.61; *p* = 0.01). Specific patients are represented by green dots. The trend line is the green dotted line.

**Figure 21:**
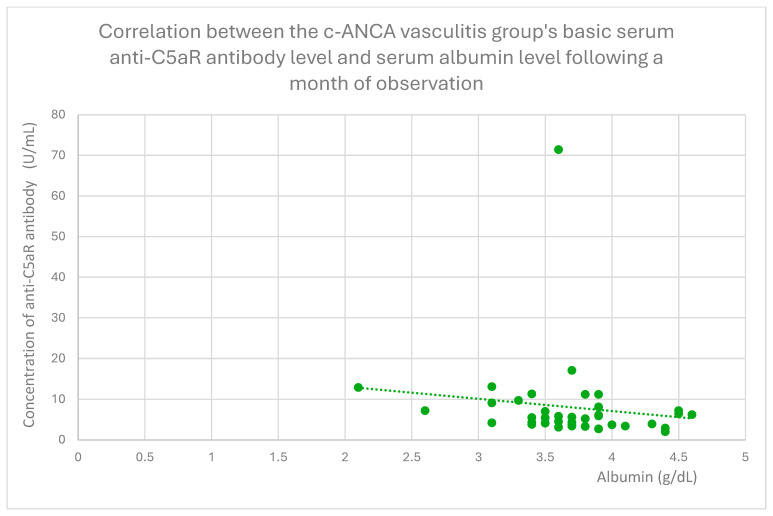
Correlation between the c-ANCA vasculitis group’s basic serum anti-C5aR antibody level and serum albumin level following a month of observation (r = −0.33; *p* = 0.04). Specific patients are represented by green dots. The trend line is the green dotted line.

Anti-C5aR baseline serum antibody levels correlated positively with ds DNA antibody levels in the lupus nephritis group (Figure 19). Additionally, following a month of monitoring, anti-C5aR antibody levels showed a positive correlation with total protein levels in the FSGS group (Figure 20) and a negative correlation with albumin levels in the c-ANCA vasculitis group (Figure 21).

### 3.6. Anti-C3aR and Anti-C5aR Antibody Level Correlation

**Figure 22:**
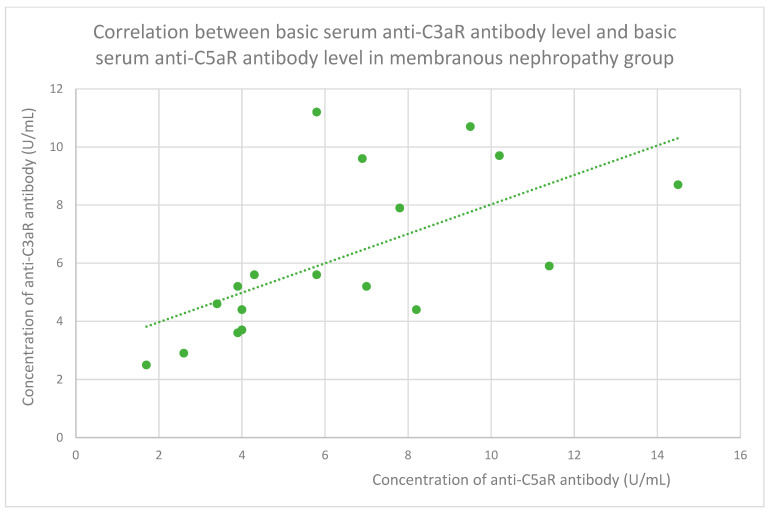
The correlation of basic serum anti-C3aR antibody level and basic serum anti-C5aR antibody level in the group of patients with membranous nephropathy (r = 0.72; *p* = 0.001). Specific patients are represented by green dots. The trend line is the green dotted line.

**Figure 23:**
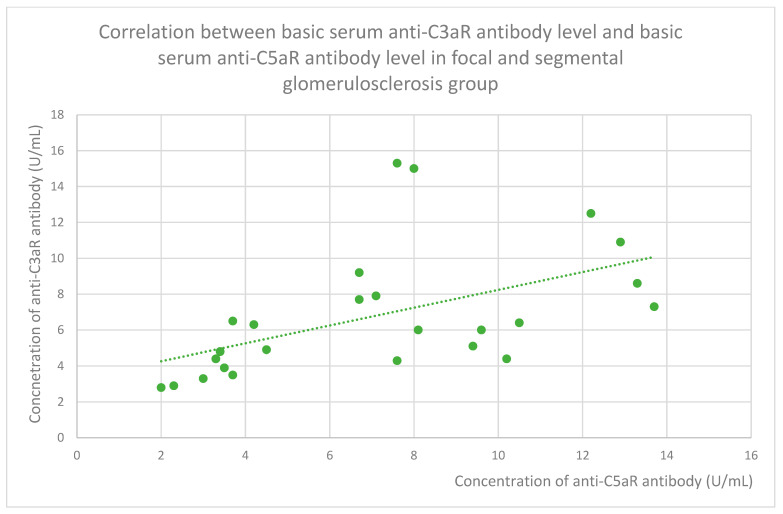
Correlation between basic serum anti-C3aR antibody level and basic serum anti-C5aR antibody level in focal and segmental glomerulosclerosis group (r = 0.64; *p* = 0.001). Specific patients are represented by green dots. The trend line is the green dotted line.

**Figure 24:**
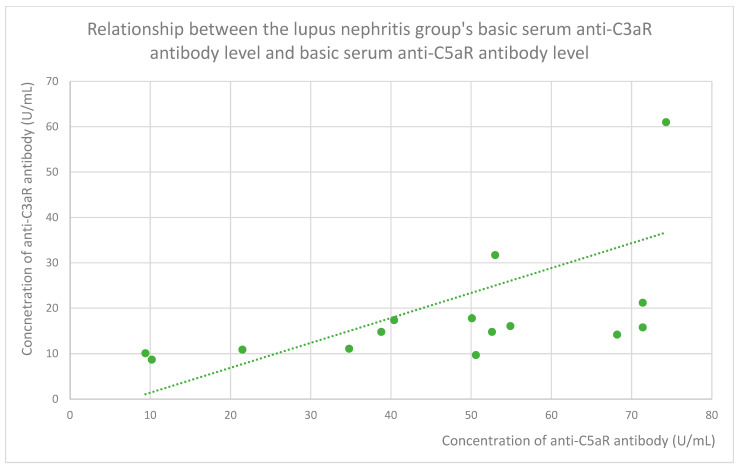
Relationship between the lupus nephritis group’s basic serum anti-C3aR antibody level and basic serum anti-C5aR antibody level (r = 0.78; *p* = 0.001). Specific patients are represented by green dots. The trend line is the green dotted line.

**Figure 25:**
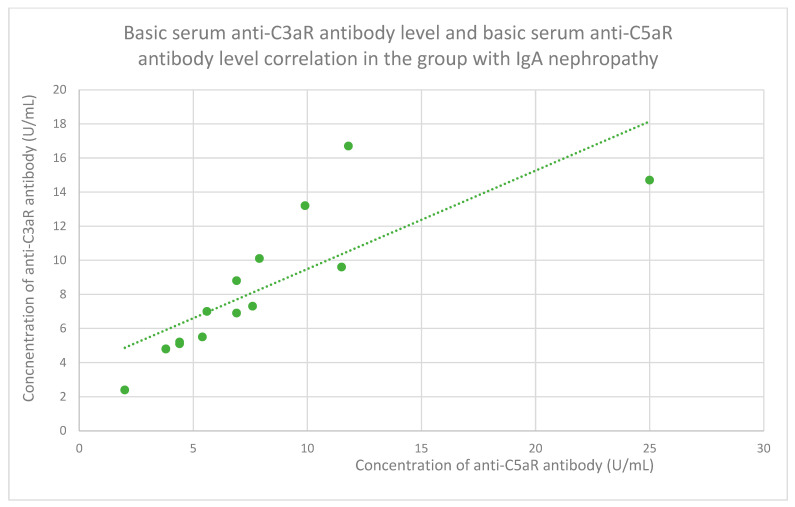
The correlation of basic serum anti-C3aR antibody level and basic serum anti-C5aR antibody level in the group with IgA nephropathy (r = 0.96; *p* = 0.001). Specific patients are represented by green dots. The trend line is the green dotted line.

**Figure 26:**
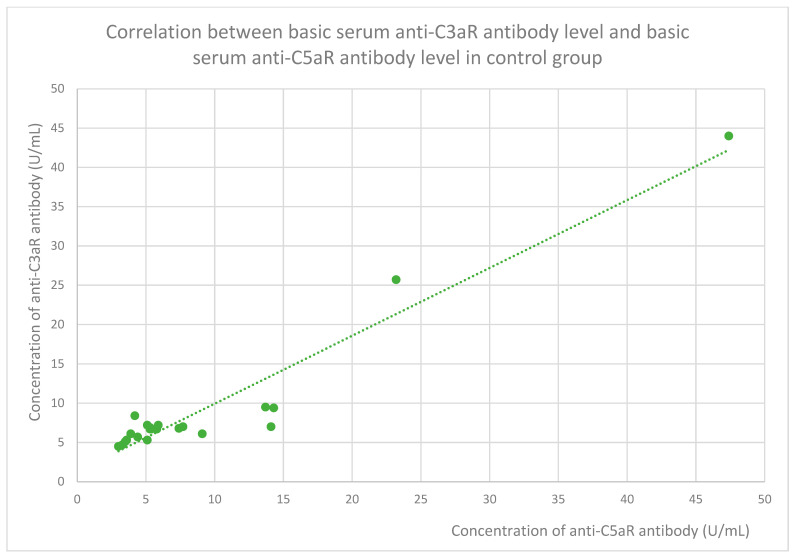
Correlation between basic serum anti-C3aR antibody level and basic serum anti-C5aR antibody level in control group (r = 0.78; *p* = 0.001). Green dots represent particular patients. Green dotted line—trend line.

**Figure 27:**
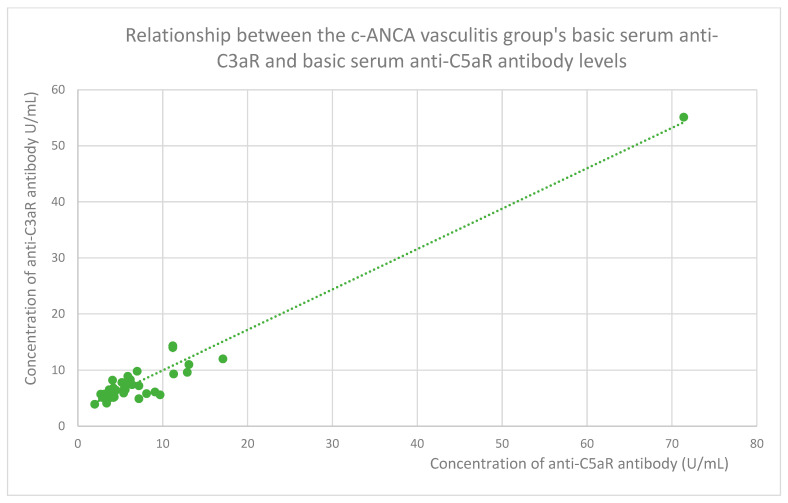
Relationship between the c-ANCA vasculitis group’s basic serum anti-C3aR and basic serum anti-C5aR antibody levels (r = 0.74; *p* = 0.001). Specific patients are represented by green dots. The trend line is the green dotted line.

**Figure 28:**
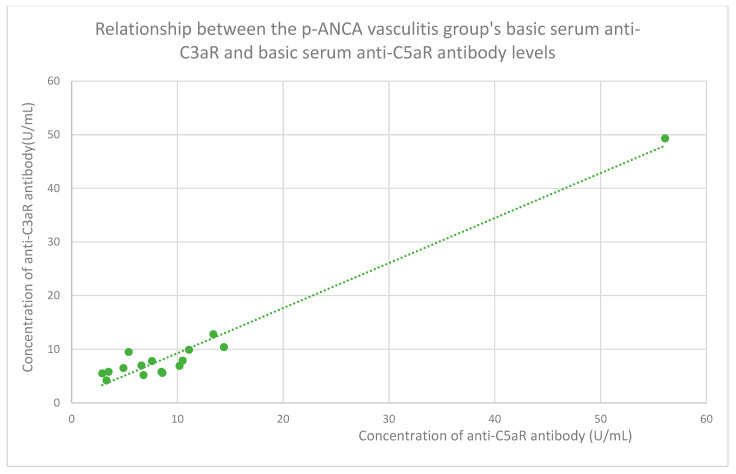
Relationship between the p-ANCA vasculitis group’s basic serum anti-C3aR and basic serum anti-C5aR antibody levels (r = 0.75; *p* = 0.001). Specific patients are represented by green dots. The trend line is the green dotted line.

The membranous nephropathy group (Figure 22), focal and segmental glomerulosclerosis group (Figure 23), lupus nephritis group (Figure 24), IgA nephropathy group (Figure 25), control group (Figure 26), c-ANCA vasculitis group (Figure 27), and p-ANCA vasculitis group (Figure 28) all showed positive correlations between anti-C3aR antibody levels and anti-C5aR antibody levels.

## 4. Discussion

Compared to the groups of patients with various glomerular diseases and the healthy controls, the lupus nephritis patients had greater levels of both anti-C3aR and anti-C5aR antibodies (Figure 2 and Figure 3). Moreover, anti-C3aR antibodies correlated negatively at many time points of observation with albumin and total protein levels in the lupus nephritis group (Figure 4, Figure 5, Figure 6, Figure 7, Figure 8, Figure 9 and Figure 10). Higher levels of anti-C3aR seem to be related to lower levels of albumin and total protein, so they reflect some components of disease activity.

Additionally, this correlation continues throughout many timepoints, so it seems to support the thesis about the predictive value of anti-C3aR antibodies in terms of total protein and albumin levels in lupus nephritis.

We did not find correlations with creatinine, but the patients during the observation period were treated, so we suppose that their treatment prevented kidney function deterioration, despite having active lupus nephritis.

Moreover, we found a significant correlation between both anti-C3aR (Figure 11) and anti-C5aR (Figure 19) antibody levels and dsDNA antibody levels in lupus nephritis. This is additional data supporting the thesis that the levels of these antibodies are related to lupus nephritis activity.

These results are not very surprising, taking into account that both C3aR [13] and C5aR [25] signaling was found to be involved in the pathogenesis of lupus nephritis. To date, apart from our study, there have been no other studies evaluating anti-C3aR and anti-C5aR antibody predictive values in lupus nephritis. The only study [41] that evaluated anti-C5aR antibody levels in systemic lupus erythematosus was, in fact, a study of c-ANCA vasculitis, and patients with systemic lupus erythematosus were one of the control groups in that study. The authors of that study [41] found that ant-C5aR antibody levels were lower in c-ANCA vasculitis patients compared to healthy controls and systemic lupus erythematosus patients.

Levels of anti-C5aR in systemic lupus erythematosus patients in that study [41] were not different from healthy controls. However, patients with lupus in that study [41] were patients with different organ manifestations of systemic lupus erythematosus that did not necessarily involve the kidneys. Our patients had lupus nephritis with active proteinuria, which is recognized as strong systemic lupus erythematosus activation. Moreover, our findings provide stronger data for the connection between anti-C3aR and lupus nephritis activity than anti-C5aR and lupus nephritis activity. Overall, our data indicate that anti-C3aR antibody levels are connected to lupus nephritis and active proteinuria activity. Our findings are consistent with observations that C3aR expression in the immunohistochemical staining of kidney specimens is more pronounced in cases of active lupus nephritis [13]. Lupus nephritis is a very active immunological disease associated with the creation of different autoantibodies, so we posit that anti-C3aR and anti-C5aR antibodies may appear as part of the lupus autoantibody spectrum.

Another finding from our study was a positive correlation between anti-C3aR antibody level and total protein level at different time points of observation, and additionally between anti-C3aR antibody levels and basic albumin in the focal and segmental glomerulosclerosis group (Figure 12, Figure 13, Figure 14, Figure 15 and Figure 16). These data are weaker than the data for lupus nephritis, because there was no difference between anti-C3aR antibody levels in the focal and segmental glomerulosclerosis group and the control group.

Nevertheless, we still have a series of consistent data. Albumin levels at different time points of observation were also close to statistical significance, and in the case of a bigger group of patients, these data would probably also achieve statistical significance. In the case of anti-C5aR antibody levels, we found a significant positive correlation between the level of these antibodies and total protein in the group with focal and segmental glomerulosclerosis after a month of monitoring them (Figure 20).

There have been two published studies [12,18] suggesting the role of C3aR in focal and segmental glomerulosclerosis pathogenesis and two studies supporting the role of C5aR [28,29] in FSGS pathogenesis. Up to date, there have been no studies evaluating anti-C3aR and anti-C5aR in focal and segmental glomerulosclerosis. The results of our study suggest some association with anti-C3aR antibody levels, but because of the small group of patients studied, these results should be treated as preliminary. There is a positive correlation between antibodies and total protein levels. This is the opposite of lupus nephritis. Higher levels of anti-C3aR antibody were connected with lower disease activity.

More advanced changes in focal and segmental glomerulosclerosis may suggest the domination of sclerotic changes and potentially fewer antigens from living cells able to promote antibody production. The earlier stages of FSGS development may be connected with greater numbers of living cells with more antigens and more pronounced active inflammation. We postulated the same mechanism in our study for the anti-PAR 1 antibody, because of a similar kind of correlation [46]. This is the only hypothesis based on the nature of focal and segmental glomerulosclerosis disease [47] and it should be checked using molecular models.

After a month of observation, we also observed a significant relationship between the levels of anti-C3aR antibodies and creatinine and total protein in the group with IgA nephropathy (Figure 17 and Figure 18). Both correlations are positive, meaning these data are not consistent, suggesting, on one hand, a connection between high antibody levels and a deterioration of kidney function and, on the other hand, an increase in total protein with higher antibody levels. The existing data about the significance of C3aR signaling in IgA nephropathy [14] suggest that C3aR blockade suppresses the development of IgA nephropathy. Anti-C3aR antibody levels may have some association with the clinical course of the disease. The inconsistency of our data about anti-C3aR’s influence on the course of the disease indicates that we should not extract definitive conclusions from these data, other than that some association is possible, and our results should be checked in studies with bigger groups of patients.

The negative correlation between anti-C5aR antibody levels and albumin levels after 1 month of observation in the cANCA vasculitis group (Figure 21) is consistent with other authors’ study results [41], suggesting the diminishment of anti-C5aR antibody levels in case of c-ANCA vasculitis activation.

Anti-C3aR and anti-C5aR antibody levels correlate with each other statistically significantly in all groups (Figure 22, Figure 23, Figure 24, Figure 25, Figure 26, Figure 27 and Figure 28) apart from the mesangial proliferative (non-IgA) glomerulonephritis group. This group was the smallest of all our groups. In this group, the tendency was similar to that observed in the other groups but did not achieve statistical significance because of the small number of patients. The correlation between both kinds of antibodies was very easy to predict because antigens of these antibodies cooperate together in the complement system [48].

Anti-C3aR and anti-C5aR antibody levels are also correlated in the control group. This phenomenon probably reflects the connection between C3a and C5a in the complement system and suggests the existence of regulation control mechanisms between C3aR, C5aR, anti-C3aR, and ant-C5aR. We postulate there are similar mechanisms between anti-AT1R (anti-angiotensin 2 type 1 receptor antibody) and AT2R (anti-angiotensin 2 type 2 receptor antibody) [49]. These hypotheses should be of course checked using molecular models.

### 4.1. Study Limitations

Patients with membranous glomerulonephritis, FSGS, and non-IgA mesangial proliferative glomerulonephritis were treated with standard immunosuppressive therapy during the observation period. This included three 500 mg doses of methylprednisolone, followed by oral prednisone (1 mg/kg) with a progressive dosage reduction. Methylprednisolone (500 mg) was given to patients with IgA nephropathy three times, spaced two months apart. For six months, patients were also administered prednisone (0.5 mg/kg) every other day in between the methylprednisolone pulses. Azathioprine (100 mg) was administered daily, cyclophosphamide (500 mg) was administered every two weeks (six doses total), and a starting dose of prednisone (1 mg/kg), which was gradually decreased, was administered as part of the treatment plan for LN and vasculitis.

The control group did not differ significantly from the other groups in terms of age and sex, but the relatively small numbers of patients in all of the groups creates the risk of finding significant differences in the case of studies with a greater number of patients. There was no power analysis or sample size calculation in the analysis.

The current study’s conclusions need to be verified using a larger sample size and further assessed through the use of a prospective cohort design. Additionally, more research is required to determine the anti-C3aR and anti-C5aR antibody activity in particular glomerular disorders using molecular models. A meta-analysis of research and artificial intelligence models should be part of future efforts to fully comprehend the relationships among various indicators and antibodies in glomerular disorders.

Despite some limitations, our study also has some strengths. We found many consistent correlations that were statistically significant (even in small groups of patients) with an r value between 0.41 and 0.94, most of them 0.5–0.6. Moreover, our study is the first study in the world assessing the role of anti-C3aR and anti-C5aR antibodies in glomerular diseases.

### 4.2. Future Perspectives

The anti-C3aR antibody may serve as a valuable prognostic marker for FSGS and LN (lupus nephritis). Furthermore, it could also function as an indicator of lupus nephritis activity. Evaluating the levels of these antibodies is a simple process that requires only serum samples. Such assessments could reduce the need for kidney biopsies and may prove particularly beneficial when used in conjunction with comparable assays for additional antibodies, such as anti-CXC motif chemokine receptor 3 (CXCR3) antibody, anti- endothelin A receptor (ETAR) antibody [50], anti-proteinase-activated receptor 1 PAR1 antibody, and anti-angiotensin 2 converting enzyme (ACE 2) antibody [46]. Future research should concentrate on the interactions between these antibodies and therapeutic agents, such as the C5a inhibitor eculizumab [51] or the C5aR inhibitor avacopan [52]. Interestingly, resveratrol was also found to ameliorate FSGS through C3aR/C5aR [28]. Nevertheless, our study represents the first investigation into the levels of anti-C3aR and anti-C5aR antibodies in glomerular diseases, revealing an association between the clinical development of lupus nephropathy and focal and segmental glomerulosclerosis and antibody levels.

## 5. Conclusions

Anti-C3aR and anti-C5aR antibodies are higher in lupus nephritis patients compared to other glomerulonephritis patients and healthy individuals.

Albumin and total protein levels appear to be correlated positively with anti-C3aR antibody levels in FSGS and negatively in lupus nephritis patients.

## 6. Disclosures

The study was partially funded by CELLTREND (Luckenwalde, Germany) according to the authors. Blood biochemical analyses were provided at no cost by CELLTREND (Luckenwalde, Germany).

The company CELL-TREND (Luckenwalde, Germany) employed Kai Schulze-Forster and Harald Heidecke. The other authors affirm that no financial or commercial ties that might be interpreted as a potential conflict of interest existed when the study was carried out.

## Figures and Tables

**Table 1:** Clinical parameters of patients with various glomerular disorders.

Specific Glomerular Disorder	Basic Level of Creatinine in Serum (mg/dL)	Basic Estimated Glomerular Filtration Rate (mL/min/1.73m^2^) MDRD (Modification Diet in Renal Disease)	Basic Blood Urea Nitrogen (mg/dL)	Albumin-to- Creatinine Ratio	Proteinuria (mg/per Day)	Basic Level of Total Protein in Serum (g/dL)	Basic Level of Albumin in Serum (g/dL)
membranous glomerulonephritis (*n* = 18)	1.25 (0.8–3.3)	66 (15–106)	12 (8–32)	1.6 (0.3–7.1)	2640 (100–15,800)	4.8 (3.7–5.9)	2.8 (1.7–3.9)
focal and segmental glomerulosclerosis (*n* = 25)	1.21 (0.73–3.19)	68 (26–126)	12 (8–30)	1.3 (0.3–7.5)	2300 (70–13,990)	5 (3–7.3)	2.9 (1.3–4.8)
lupus nephritis (*n* = 17)	1.06 (0.77–2.19)	68 (31–116)	9 (7–23)	0.8 (0.3–3.1)	1590 (180–5950)	5.5 (3.8–7.3)	3.1 (2–4)
IgA nephropathy (*n* = 14)	1.06 (0.71–1.82)	70 (35–131)	9.5 (7–20)	0.6 (0.3–2.2)	940 (90–4540)	5.65 (4.4–6.5)	3.4 (2.2–4)
mesangial proliferative (non-IgA) glomerulonephritis (*n* = 6)	0.93 (0.59–1.55)	105 (40–131)	8.5 (6–16)	0.8 (0.4–2.9)	2580 (620–7130)	4.8 (3.9–5.2)	2.8 (1.6–3.2)
control group (*n* = 22)	1.2 (0.9–1.3)	63 (60–78)	12 (9–16)	0 (0–0)	0 (0–0)	7.4 (6.6–8.2)	4.4 (3.5–5.2)
c-ANCA vasculitis (*n* = 40)	1.81 (0.69–7.78)	45 (7–126)	19.8 (7–75)	0.4 (0.3–10.9)	640 (60–19,000)	6.3 (5.3–7.1)	3.6 (2.4–4.6)
p-ANCA vasculitis (*n* = 16)	3.13 (0.79–9.04)	19 (5–93)	30 (8–81)	0.8 (0.3–5)	1730 (140–12,300)	5.95 (4.8–8.3)	3.5 (2.8–4.3)

Legend: Values in table are medians followed by ranges (ranges are presented in parentheses) of clinical parameters in specific groups.

**Table 2:** Clinical parameters of patients with various glomerular disorders.

Specific Glomerular Disorder	Age (Years)	Sex (Ratio of Men/Women)	Hemoglobin (g/dL)	HCT (Hematocrit) (%)	Leukocytes (Per Microliter)
membranous glomerulonephritis (*n* = 18)	51.5 (28–69)	1.22	13.5 (11.3–16)	44.5 (38–52.6)	6715 (2500–10,780)
focal and segmental glomerulosclerosis (*n* = 25)	48 (19–74)	1.27	13.9 (9.3–18)	45.7 (36.8–55.2)	7690 (4130–10,500)
lupus nephritis (*n* = 17)	34 (19–66)	0.89	13.2 (10.5–17.3)	43 (34.5–56.9)	6600 (4090–10,900)
IgA nephropathy (*n* = 14)	45.5 (20–60)	1	14.8 (12.2–16.5)	48.6 (46–54.3)	8420 (5360–10,900)
Mesangial proliferative (non-IgA) glomerulonephritis (*n* = 6)	28 (20–52)	1	14.8 (10.1–18)	45.2 (33.2–55)	10,200 (6200–10,490)
control group (*n* = 22)	44 (26–80)	1	14.6 (12.3–16.9)	45.7 (37.9–53)	6270 (4280–7780)
c-ANCA vasculitis (*n* = 40)	58 (21–81)	0.82	12 (7.7–16.1)	37.3 (27–49.7)	6555 (3740–10,450)
p-ANCA vasculitis (*n* = 16)	62 (37–87)	1.27	9.6 (8.3–13.2)	30.4 (28.4–42)	4905 (3240–9620)

Legend: Values in table are medians followed by ranges (ranges are presented in parentheses) of clinical parameters in specific groups.

## Data Availability

The article and Supplementary Materials contain the data and the original contributions made by this study. Please contact the corresponding author if you have further questions.

## Supplementary Materials

The following supporting information can be downloaded at: https://www.mdpi.com/article/10.3390/jcm14176082/s1, Values of serum anti-C3aR antibody levels in particular patient groups (U/mL) are shown in Figure S1. Values of serum anti-C5aR antibody levels in particular patient groups (U/mL) are shown in Figure S2.

## Abbreviations

The following abbreviations are used in this manuscript:

ANAs	antinuclear antibodies
ANOVA	analysis of variance
Anti-ACE2	anti-angiotensin 2 converting enzyme antibody
Anti-AT1R	anti-angiotensin 2 type 1 receptor antibody
Anti-AT2R	anti-angiotensin 2 type 2 receptor antibody
Anti-CXCR3	anti-C-X-C motif chemokine receptor 3 antibody
Anti-ETAR	anti endothelin A receptor antibody
Anti-PAR1	anti proteinase-activated receptor type 1 antibody
cANCA	cytoplasmic anti-neutrophil cytoplasmic antibody
c3aR	complement 3a factor receptor
c5aR	complement 5a factor receptor
dsDNA	anti-double-stranded deoxyribonucleic acid antibody
eGFR	estimated glomerular filtration rate
ELISA	enzyme-linked immunosorbent assay
FSGS	focal and segmental glomerulosclerosis
GPCR	G-protein-coupled receptor
HCT	hematocrit
HLA	human leukocyte antigens
IgA	immunoglobulin A
IL-6	interleukin 6
IU	international unit
LN	lupus nephritis
MCP 1	monocyte chemoattractant protein 1
MDRD	Modification Diet in Renal Disease
m-RNA	mitochondrial ribonucleic acid
pANCA	perinuclear anti-neutrophil cytoplasmic antibody
POD	horseradish peroxidase
Th1	T helper lymphocytes type 1
TMB	tetramethylbenzidine
U	unit
