# Supplementary figures and images for: The Influence of Anti-C3aR and Anti-C5aR Antibody Levels on the Course of Specific Glomerulonephritis Types
Source: J Clin Med. 2025 Aug 28;14(17):6082. doi: 10.3390/jcm14176082 (PMC12429157; doi:10.3390/jcm14176082)

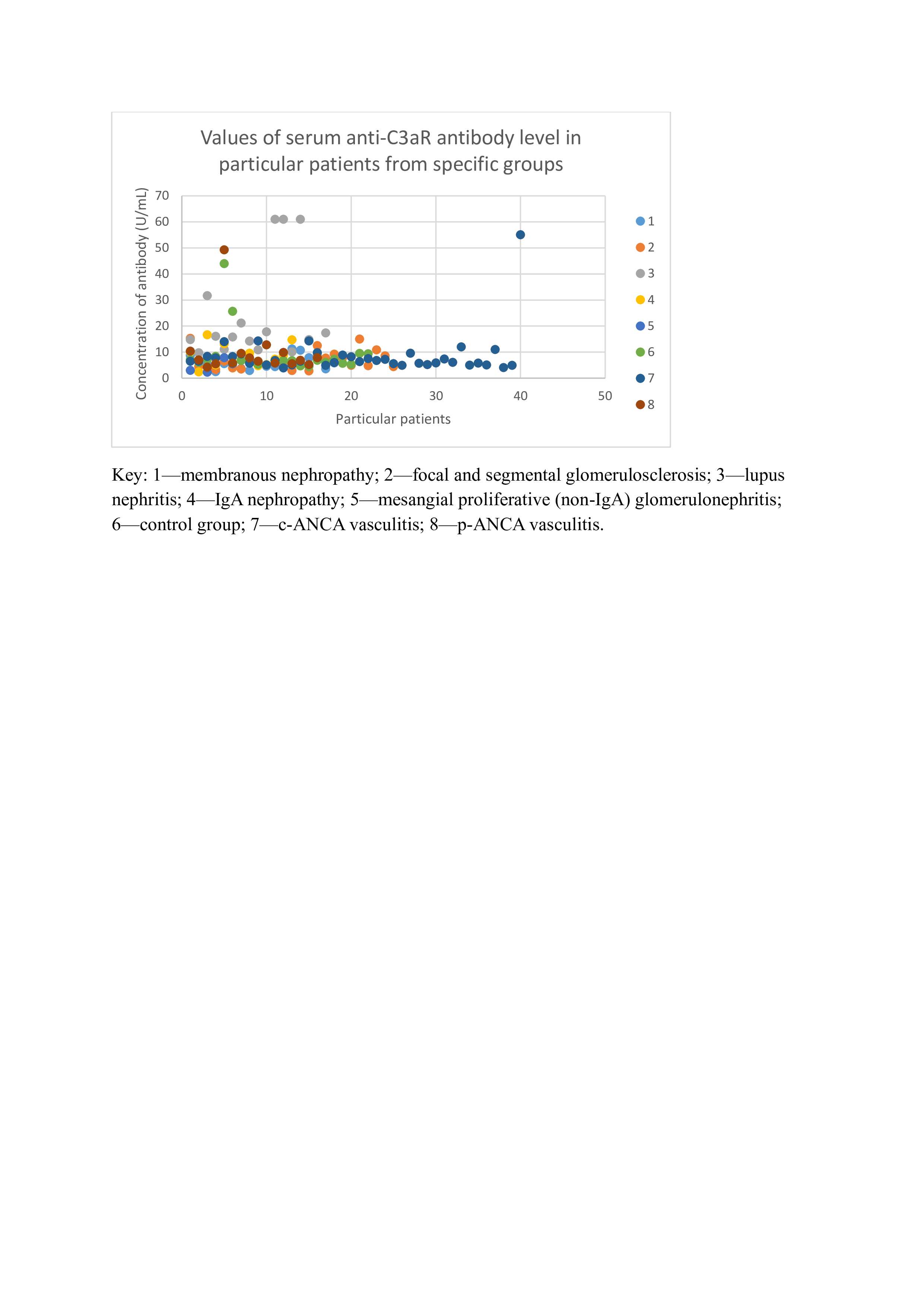

Supplement: Supplementary file 1 [file jcm-14-06082-s001.zip › Figure S1.jpg]

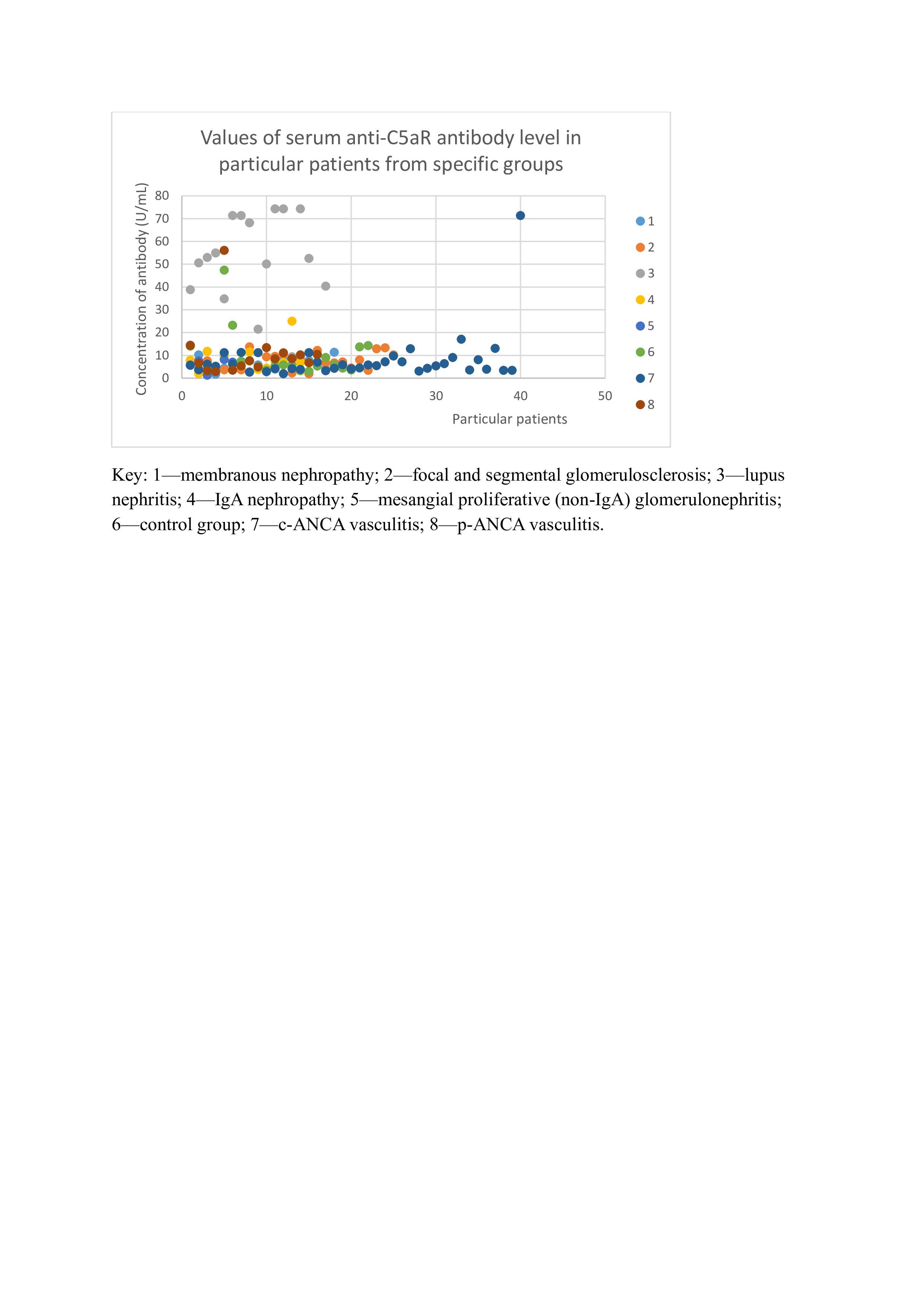

Supplement: Supplementary file 1 [file jcm-14-06082-s001.zip › Figure S2.jpg]
